# Inhibition of microRNA-660-5p decreases breast cancer progression through direct targeting of TMEM41B

**DOI:** 10.1186/s41065-024-00357-5

**Published:** 2024-12-21

**Authors:** Valeria Villarreal-García, José Roberto Estupiñan-Jiménez, Vianey Gonzalez-Villasana, Pablo E. Vivas-Mejía, Marienid Flores-Colón, Irma Estefanía Ancira-Moreno, Patricio Adrián Zapata-Morín, Claudia Altamirano-Torres, José Manuel Vázquez-Guillen, Cristina Rodríguez-Padilla, Recep Bayraktar, Mohamed H. Rashed, Cristina Ivan, Gabriel Lopez-Berestein, Diana Reséndez-Pérez

**Affiliations:** 1 Facultad de Ciencias Biológicas, Departamento de Biología Celular y Genética, Universidad Autónoma de Nuevo León, San Nicolás de los Garza, Nuevo León, México; 2 Department of Biochemistry, Medical Sciences Campus, University of Puerto Rico, San Juan, Puerto Rico; 3 Comprehensive Cancer Center, Medical Sciences Campus, University of Puerto Rico, San Juan, Puerto Rico; 4 Facultad de Ciencias Biológicas, Laboratorio de Micología y Fitopatología, Unidad de Manipulación Genética, Universidad Autónoma de Nuevo León, San Nicolás de los Garza, Nuevo León, México; 5 Facultad de Ciencias Biológicas, Laboratorio de Inmunología y Virología, Universidad Autónoma de Nuevo León, San Nicolás de los Garza, Nuevo León, México; 6 Department of Translational Molecular Pathology, The University of Texas MD Anderson Cancer Center, Houston, TX USA; 7 Clinical Pharmacy Department, Faculty of Pharmacy (Boys), Al-Azhar University, Cairo, Egypt; 8 Department of Experimental Therapeutics, The University of Texas MD Anderson Cancer Center, Houston, TX USA

**Keywords:** Breast Cancer, Cancer Progression, MicroRNAs, miR-660-5p, Transmembrane Protein 41B

## Abstract

**Background:**

Breast cancer is the most prevalent cancer among women worldwide. Most breast cancer-related deaths result from metastasis and drug resistance. Novel therapies are imperative for targeting metastatic and drug-resistant breast cancer cells. Accumulating evidence suggests that dysregulated microRNAs (miRNAs) promote breast cancer progression, metastasis, and drug resistance. Compared with healthy breast tissue, miR-660-5p is notably overexpressed in breast cancer tumor tissues. However, the downstream effectors of miR-660-5p in breast cancer cells have not been fully elucidated. Our aim was to investigate the role of miR-660-5p in breast cancer cell proliferation, migration, invasion, and angiogenesis and to identify its potential targets.

**Results:**

Our findings revealed significant upregulation of miR-660-5p in MDA-MB-231 and MCF-7 cells compared with MCF-10 A cells. Furthermore, inhibiting miR-660-5p led to notable decreases in the proliferation, migration, and invasion of breast cancer cells, as well as angiogenesis, in HUVEC cells. Through bioinformatics analysis, we identified 15 potential targets of miR-660-5p. We validated TMEM41B as a direct target of miR-660-5p via Western blot and dual-luciferase reporter assays.

**Conclusions:**

Our study highlights the upregulation and involvement of miR-660-5p in breast cancer cell proliferation, migration, invasion, and angiogenesis. Additionally, we identified TMEM41B as a direct target of miR-660-5p in breast cancer cells.

**Supplementary Information:**

The online version contains supplementary material available at 10.1186/s41065-024-00357-5.

## Background

Breast cancer ranks among the most frequently diagnosed cancers in women globally, with an estimated 2.3 million cases in 2022, and continues to be the leading cause of cancer-related death among women [1, 2]. This disease is highly heterogeneous, exhibiting diverse biological, morphological, and molecular characteristics, which contribute to varying clinical outcomes and responses to treatment [3]. According to the molecular classification of breast cancer, which categorizes tumors on the basis of the presence of hormonal receptors (estrogen and progesterone) and human epidermal growth factor receptor 2 (HER2) expression, the luminal A subtype is predominant in the United States, affecting 66% of patients, followed by the luminal B (10%), basal or triple-negative (10%), and HER2-positive (4%) subtypes [4]. Like many cancer types, the majority of breast cancer-related deaths are attributed to metastasis and drug resistance. Therefore, there is an urgent need for novel therapies targeting highly metastatic and drug-resistant cancer cells.

MicroRNAs (miRNAs) are small noncoding RNAs that play crucial roles in various biological processes [5]. They regulate gene expression posttranscriptionally by binding to the 3’-untranslated region (3′ UTR) of target mRNAs, leading to mRNA degradation or translational repression [6]. Dysregulation of miRNAs is common in many cancers, including breast cancer, and contributes to disease progression, metastasis, and drug resistance [7, 8]. Depending on their expression levels and the functions of their targets, miRNAs can act as oncogenes or tumor suppressors [7].

MiR-660-5p has been found to be significantly upregulated in breast cancer patients [9–12] and cell lines [12–14]. It has been suggested as a potential prognostic marker in breast cancer [10]. High expression of miR-660-5p in breast cancer cells indicates its role in promoting proliferation and metastasis while suppressing apoptosis through direct binding to the transcription factor CP2 (TFCP2) [13], downregulation of tet-eleven translocation 2 (TET2), and activation of the PI3K/AKT/mTOR signaling pathway [14]. However, recent bioinformatic analyses of updated miRNA databases suggest that miR-660-5p may regulate other genes not previously described in breast cancer cells.

In this study, we investigated the biological effects of targeting miR-660-5p on the proliferation, migration, invasion, and angiogenesis of breast cancer cells. Through comprehensive analysis of publicly available miRNA target prediction databases, we identified TMEM41B as a novel direct target of miR-660-5p. Survival analysis revealed associations of both miR-660-5p and TMEM41B with overall survival in breast cancer patients.

## Methods

### Cells and culture conditions

The human breast epithelial cell line MCF-10 A; the human breast cancer cell lines MDA-MB-231 and MCF-7; and the human umbilical vein endothelial cell line HUVEC were purchased from the American Type Culture Collection (ATCC). MCF-10 A cells were maintained in mammary epithelial cell growth medium (MEBM) supplemented with bovine pituitary extract (BPE), human epidermal growth factor (hEGF), insulin, hydrocortisone, and gentamicin sulfate amphotericin B (GA-1000) (Lonza, Switzerland). For the remaining cell lines, Dulbecco’s modified Eagle medium (DMEM)/Nutrient mixture F-12 ham medium (Sigma Aldrich-Merck, USA) supplemented with 10% fetal bovine serum (GenClone, USA) and 1% antibiotics (Gibco, USA) was used. All the cell lines were cultured at 37 °C in a 5% CO_2_ atmosphere. Experiments were performed at 60–80% cell confluency.

### RNA isolation and quantitative real-time polymerase chain reaction (RT‒qPCR)

Total RNA from MCF-10A, MCF-7, and MDA-MB-231 cells was extracted via the miRNeasy Mini Kit™ (Qiagen, Germany) according to the manufacturer’s instructions. The RNA concentration was determined via a Thermo Scientific Nanodrop spectrophotometer. RNA samples with an optical density A260/280 ratio between 1.8 and 2.1 were used for qRT‒PCR. qRT‒PCR was performed with the MystiCq™ MicroRNA™ Quantitation System (Sigma Aldrich-Merck). Briefly, cDNA was synthesized with the MystiCq™ microRNA cDNA Synthesis Mix Kit according to the manufacturer’s instructions. RT‒qPCR was performed with 10 µM MystiCq Universal PCR Primer (Sigma Aldrich-Merck), 10 µM miR-660-5p primer (5’-CCATTGCATATCGGAGTTGAA-3’) (IDT, USA) or SNORD44 primer (5´ GCAAATGCTGACTGAACATGAA 3’) (IDT). PCRs were performed in a Roche Light Cycler Nano (Roche Diagnostics, Germany) instrument using the following cycling conditions: 95 °C for 2 min followed by 40 cycles of 5 s at 95 °C and 30 s at 60 °C. The comparative cycle threshold (ΔΔCT) [15] was used to quantify the expression of miR-660-5p, and SNORD44 was used as the endogenous control. The miR-660-5p levels in the breast cancer cell lines represented an n-fold difference in the miRNA transcript levels in the samples compared with those in the control MCF-10 A cells.

### Cell transfections

MCF-7 cells (3.0 × 10^5^), MDA-MB-231 cells and HUVEC cells (9.0 × 10^4^) were seeded in 6-well plates. Twenty-four hours later, the cells were transfected with the miR-660-5p inhibitor or miRNA inhibitor negative control (NC) (Invitrogen) (100 nM final concentration) via HiPerFect (Qiagen) as a transfection agent at a 1:3 ratio (miR-660-5p inhibitor: HiPerFect) and Opti-MEM (Gibco). The transfection mixture was incubated for 20 min at room temperature. The cell culture media was replaced with a transfection mixture that was added dropwise. Transfection was carried out for 24 h, after which the cell pellets were collected for subsequent experiments. Transfected cells were subjected to functional assays, RT‒qPCR, and Western blotting.

### Clonogenic assay

Breast cancer cells were transfected as described above. Twenty-four hours after transfection, MDA-MB-231 cells (400 cells) and MCF-7 cells (800 cells) were seeded into 6-well plates. After 10 days of incubation for the MDA-MB-231 cells and 18 days for the MCF-7 cells, the colonies were stained with 0.5% crystal violet in methanol. Colonies containing at least 50 cells were quantified using a Zeiss Primovert inverted cell culture microscope at 10x magnification.

### Cell migration and invasion assays

Breast cancer cells were transfected as described above. Twenty-four hours after transfection, MDA-MB-231 cells (2.1 × 10^4^ cells in 200 µL of serum-free medium) and MCF-7 cells (1.0 × 10^5^ cells in 200 µL of serum-free medium) were seeded into transwell chambers (8 µM pore size, Corning, USA) coated with gelatin (100 µL) for migration and chambers coated with CultrexTM Basement Membrane Extract (BME) (R&D Systems, USA) (100 µL) for invasion assays. The wells of the 24-well plate were filled with 700 µL of complete culture medium. The incubation time for each assay varied depending on the cell line: 24 h for MDA-MB-231 cells and 72 h for MCF-7 cells for migration and 48 h for MDA-MB-231 cells and 72 h for MCF-7 cells for invasion. Following incubation, the cells were fixed and stained using the Fisher HealthCare PROTOCOL Hema 3 Manual Staining System (Fisher Scientific, USA). Images of migratory and invading cells were taken and counted in seven random fields at 40x magnification using a Leica CME microscope. The percentages of migrated and invaded cells were calculated, with the values of untreated cells considered 100% cell migration and/or invasion.

### Angiogenesis assay

HUVEC cells were transfected as described above. The cells (2.5 × 10^4^ cells/100 µL) were seeded in a 96-well plate previously covered with *Cultrex*™ Basement Membrane Extract (*BME*) (R&D Systems) (60 µL). The plate was incubated for 24 h, and pictures of four fields (10x) were taken with a Zeiss Primovert inverted cell culture microscope to observe loop formation. The number of loops per image was quantified.

### MiR-660-5p target prediction

We used nine databases (TargetScan, miRDB, RNACentral, RNA22, miSTAR, DIANA, miRmap, miRabel, and MicroT) to predict potential miR-660-5p targets. We selected the top 100 miR-660-5p target genes with the highest scores in each database. Then, we selected genes that appeared in at least three of the databases. To simplify these results, we subsequently performed an analysis using the Kaplan‒Meier plotter, in which we evaluated the expression of each gene in overall survival (OS), relapse-free survival (RFS), distant metastasis-free survival (DMFS), and the palliative performance scale (PPS), selecting the genes with significant *p*-values (*p* < 0.05).

### RT‒qPCR to validate the predicted miR-660-5p targets

A custom-made 384-well plate containing predesigned forward and reverse primers for each of the selected miR-660-5p target genes was purchased from Bio-Rad (Hercules, CA, USA). MDA-MB-231 cells (9.0 × 10^4^) were seeded in a 6-well plate and transfected with the miR-660-5p inhibitor (200 nM final concentration). Twenty-four hours later, total RNA was isolated using the GenElute Mammalian Total RNA Mini Kit (Millipore-Sigma, USA) according to the manufacturer’s instructions. The RNA was reverse transcribed via the iScript Reverse Transcription Supermix for RT‒qPCR from Bio-Rad. SYBR Green-based qPCR was performed using SsoAdvanced™ Universal SYBR^®^ Green Supermix (Bio-Rad) and a CFX384 Touch Real-Time PCR detection system. Fold changes and cycle threshold (Ct) values were calculated by the instrument’s internal software relative to MDA-MB-231 cells and normalized to β-actin along with controls for gDNA, PCR, RT, and RNA quality.

To create the volcano plot, we used the R environment (version 4.2.2) along with the ggplot2 package. The methodology involved first preprocessing the qPCR data to calculate 2-∆∆CT fold changes and *p*-values. Using ggplot, we plotted the negative log of the *p*-value against the fold change, applying a threshold line at *p* = 0.05 for statistical significance. The upregulated and downregulated genes were distinguished by their fold change values, with custom color coding for visual emphasis.

### Western blot analysis

Breast cancer cells were transfected as described above at a final concentration of 200 nM. The cells were collected and washed with 1 mL of PBS supplemented with 10 µL of protease inhibitor cocktail (Sigma Aldrich-Merck). The cell lysates were prepared with 100 µL of lysis buffer with 1 µL of protease inhibitor cocktail (Sigma Aldrich-Merck), incubated on ice for 30 min and vortexed every 10 min. The lysates were centrifuged, the supernatants were collected, and the total protein concentration was determined via Bradford’s reagent (Sigma Aldrich-Merck) following the manufacturer’s instructions. Equal amounts of each protein sample (40 µg per well) were separated by SDS‒PAGE, transferred to nitrocellulose membranes, blocked for 1 h with Blotto, a nonfat dry milk (Santa Cruz Biotechnology, USA) at 5%, and then washed and probed with the primary antibody for 24 h at 4 °C. The membranes were subsequently rinsed and incubated with the corresponding HRP-conjugated secondary antibody. Bound antibodies were detected using the ImmunoCruz Western Blotting Reagent Luminol (Santa Cruz Biotechnology). The following primary antibodies were used: TMEM41B (1:1000, cat. #68071, Cell Signaling Technology, USA) and β-actin (1:1000, cat. # sc-47778, Santa Cruz Biotechnology). The following secondary antibodies were used: goat anti-rabbit and anti-mouse IgG (H + L) horseradish peroxidase (HRP) (1:1000, cat. #31460, #31430, Invitrogen). Images of the bands were taken, and the ImageJ program was used for densitometric analysis. The results were normalized to the loading control (β-actin).

### Dual-luciferase reporter assays

MDA-MB-231 cells (7.0 × 10^3^ cells) were seeded into 96-well plates, and 24 h later, 1.5 µg of the miRNA 3’UTR target expression clone for human TMEM41B (NM_015012.3) (HmiT102502-MT06) or the miRNA target control vector for pEZX-MT06 (CmiT000001-MT06) (GeneCopoeia, USA) was transfected using HiPerFect at a 1:3 (v/v) ratio (DNA: HiPerFect) in Opti-MEM media. Six hours after plasmid transfection, the cells were transfected with inhibitor or inhibitor NC (200 nM final concentration) overnight, as described above. The vectors used included the 3’UTR of TMEM41B and the control vector. The transfection mixture included the 3’UTR vector, HiPerFect (1:3 ratio, v/v) and Opti-MEM. The next day, the media was changed to fresh DMEM/F-12 (10% FBS and 1% penicillin/streptomycin). After 48 h, the firefly and Renilla luciferase activities were measured using the Dual-Luciferase Reporter Assay System Kit (Promega, USA) according to the manufacturer’s instructions. Luminescence measurements were performed on a SynergyTM HT plate reader (BioTek^®^ Instruments). Firefly luciferase activity was normalized to that of the Renilla control. The luciferase activity was plotted relative to that of the negative 3’UTR control vector.

### Interrogation of patient survival databases

To elucidate the clinical significance of miR-660-5p and TMEM41B expression, we performed a Kaplan‒Meier survival analysis using publicly available datasets (miRpower for breast cancer and KM for breast cancer) via Kaplan‒Meier (KM) plotter (https://www.kmplot.com). By selecting hsa-miR-660, breast cancer patients were split into high- and low-expression groups according to the best cutoff values of miRNA expression determined by the best performing threshold between the lower and upper quartiles. A KM survival plot for overall survival (OS) was generated for all breast cancer patients (*n* = 1062) without any other restrictions (age, stage, grade). Similarly, we evaluated the expression levels of TMEM41B in BC patients. A KM survival plot for relapse-free survival (RFS) was generated for all breast cancer patients (*n* = 4929). *p*-values < 0.05 were considered statistically significant.

### Statistical analysis

All the experiments were performed in triplicate. Statistical analyses and graph construction were performed with GraphPad Prism software (GraphPad Software Inc., USA). *p-*values were calculated by parametric (t-test or ANOVA) analysis as determined by normality tests. *p*-values < 0.05 were considered statistically significant.

To assess the correlation between TMEM41B expression and hsa-miR-660 expression, a Spearman correlation analysis was performed. This method was chosen for its robustness in handling non-normal distributions and its reduced sensitivity to outliers. A total of 1,095 breast cancer samples were retrieved from the TCGA Cancer Atlas database using the RTCGA library (version 1.34). To focus the analysis, only tumor samples containing miRNA data were included. Data preprocessing involved filtering out missing values and retaining only unique observations. The statistical analysis was conducted using the cor.test() function from the stats library in R, and the hypothesis test evaluated whether the observed Spearman correlation coefficient (ρ\rho) significantly differed from zero. All analyses were performed using R version 4.4.2 to ensure reproducibility.

## Results

### MiR-660-5p is overexpressed in breast cancer cells

**Fig. 1:**
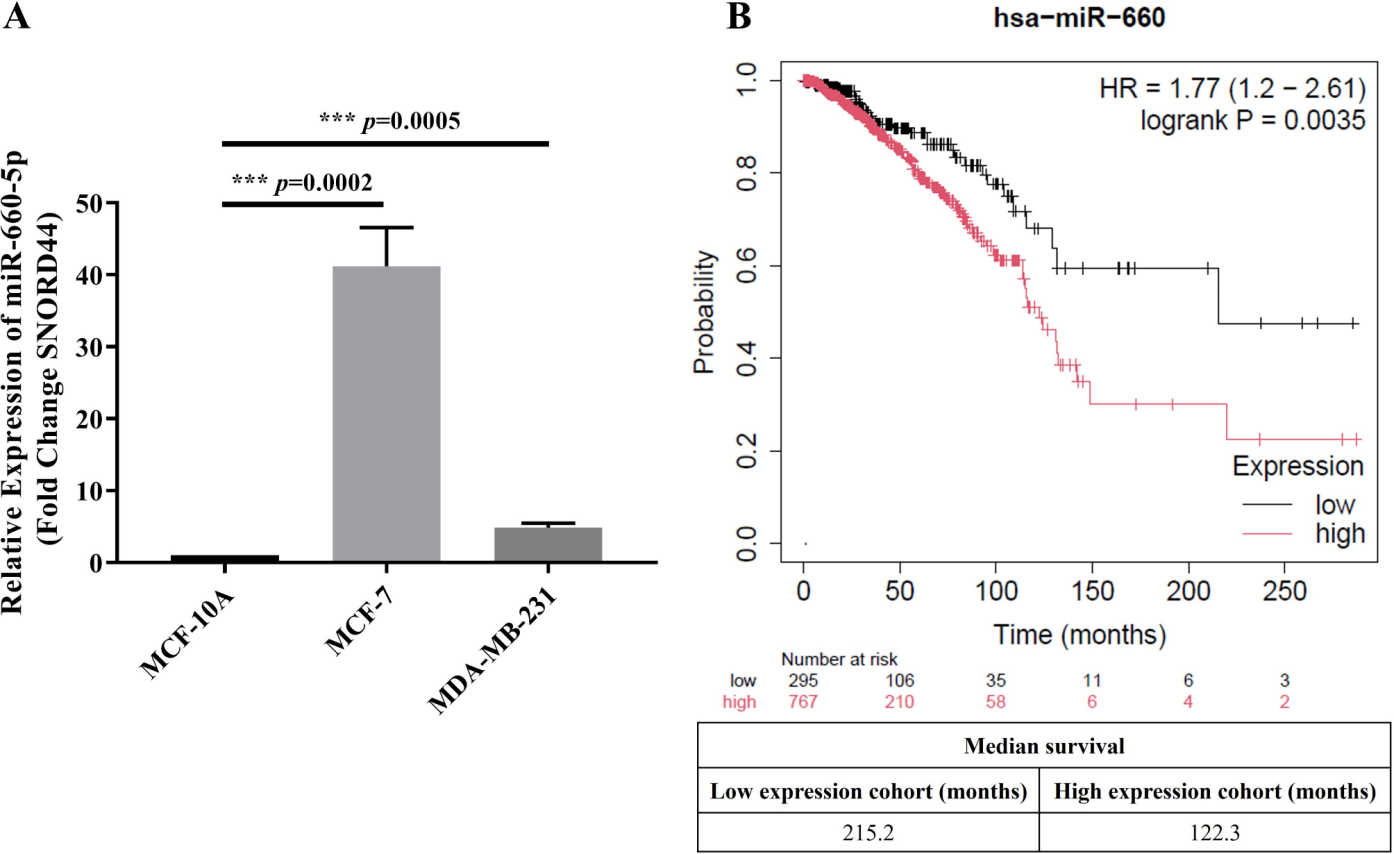
Basal levels of miR-660-5p in breast cancer cells and the clinical relevance of miR-660. **(A)** The levels of miR-660-5p were assessed by real-time PCR with specific primers for this miRNA. The experiments were performed in triplicate. Bars: means ± SDs. *** *p* < 0.001. **(B)** Kaplan‒Meier (KM) plot of the results of the miR-660 expression-based overall survival (OS) analysis. A KM plot of breast cancer patients was generated using the KM plotter searchable database (www.kmplot.com). *p*-values < 0.05 were considered statistically significant

To evaluate the effect of miR-660-5p in breast cancer, we selected the MDA-MB-231 (triple-negative) and MCF-7 (luminal A) cell lines because they represent two distinct and clinically significant breast cancer subtypes. This approach allows us to investigate the role of miR-660-5p in diverse biological contexts. MiR-660-5p expression in MDA-MB-231 and MCF-7 cells were quantified via RT‒qPCR. As depicted in Fig. 1, the miR-660-5p expression levels were markedly elevated in these breast cancer cells compared with those in the control MCF-10 A cells. Furthermore, differential expression of miR-660-5p was observed between the breast cancer cell lines, with higher levels of miR-660-5p in the MCF-7 cells than in the MDA-MB-231 cells.

To assess the clinical significance of miR-660 in breast cancer, we conducted Kaplan‒Meier analysis using the Kaplan‒Meier Plotter tool (https://kmplot.com/analysis/). Survival curves revealed a significant correlation (*p* = 0.0035) between miR-660 levels and overall survival (OS) in breast cancer patients (*n* = 1062). Patients with lower miR-660 levels had a median OS of 215.2 months, whereas those with higher miR-660 levels had a median OS of 122.3 months (Fig. 1B).

### Knockdown of miR-660-5p reduced the proliferation of breast cancer cells

**Fig. 2:**
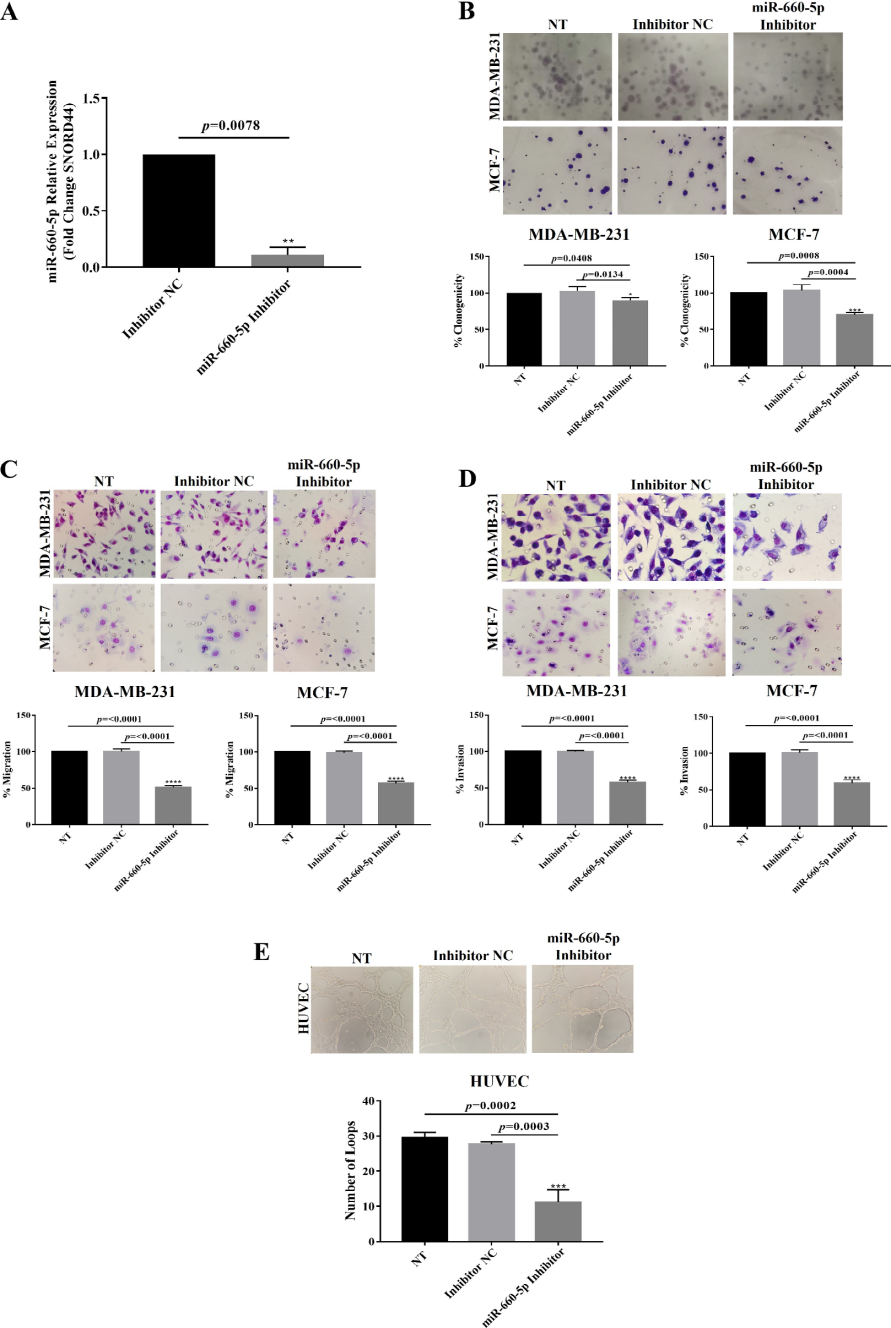
Effects of miR-660-5p knockdown on the proliferation, migration, invasion, and angiogenesis of breast cancer cells. **(A)** The efficacy of the miR-660-5p inhibitor was measured in MDA-MB-231 cells by RT‒qPCR 24 h after cell transfection. **(B)** Effects of miR-660-5p knockdown on cell proliferation **(C)**, cell migration **(D)**, cell invasion, and in vitro angiogenesis **(E)**. Transient transfections were performed as described in the “Materials and Methods” section. Images of migration, invasion, and angiogenesis were taken at 40x microscope magnification. The experiments were performed in triplicate. * *p* < 0.05; *** *p* < 0.001; **** *p* < 0.0001

To elucidate the biological role of miR-660-5p in breast cancer cells, we conducted loss-of-function assays. Compared with NC, transfection with the miR-660-5p inhibitor resulted in a significant reduction in miR-660-5p expression of approximately 90% in MDA-MB-231 cells (Fig. 2A). For the assessment of cell proliferation, colony formation assays were performed. As shown in Fig. 2B, there was a notable decrease in the number of colonies formed by both cell lines transfected with the miR-660-5p inhibitor compared with those formed by the NC-miRNA control (10% reduction in MDA-MB-231, *p* = 0.0134; 29.67% reduction in MCF-7, *p* = 0.0004). These findings indicate that the inhibition of miR-660-5p expression decreases breast cancer cell proliferation.

### Knockdown of miR-660-5p reduced the migration and invasion of breast cancer cells

Next, we investigated the role of miR-660-5p in the migration and invasion of breast cancer cells. As depicted in Fig. 2C, there was a notable decrease in the number of migrating cells in both cell lines transfected with the miR-660-5p inhibitor compared with those in the NC-miRNA control group. Specifically, cell migration was significantly reduced by 48.67% (*p* < 0.0001) in MDA-MB-231 cells and by 43.34% (*p* < 0.0001) in MCF-7 cells transfected with the miR-660-5p inhibitor. A similar trend was observed in the invasion assay (Fig. 2D), where a decrease in invasiveness was observed in both breast cancer cell lines transfected with the miR-660-5p inhibitor compared with those transfected with the NC-miRNA control. Specifically, cell invasion was significantly reduced by 42.34% (*p* < 0.0001) in MDA-MB-231 cells and by 41% (*p* < 0.0001) in MCF-7 cells transfected with the miR-660-5p inhibitor. These results suggest that inhibiting miR-660-5p can attenuate the migration and invasion of breast cancer cells, indicating a potential role in facilitating metastasis.

### Knockdown of miR-660-5p reduced in vitro angiogenesis

To assess the impact of miR-660-5p knockdown on angiogenesis, we conducted a tube formation assay. As shown in Fig. 2E, there was a substantial decrease in the number of loops formed by HUVEC cells transfected with the miR-660-5p inhibitor compared with those formed by HUVECs transfected with the NC-miRNA control (88.43% reduction, *p* = 0.0003). These results indicate that inhibiting miR-660-5p decreases angiogenesis.

### Identification of miR-660-5p target genes in breast cancer cells

To identify potential target genes of miR-660-5p in breast cancer cells, we conducted a bioinformatic analysis using nine distinct miRNA target prediction databases. We selected the top 100 targets from each database and identified genes predicted by at least three databases, resulting in 37 miR-660-5p target genes (Supplementary Table 1). To identify potential clinically relevant miR-660 target genes in breast cancer, we interrogate the Kaplan‒Meier plotter database, which assesses correlations between gene expression (mRNA, miRNA, protein) and patient outcomes across various cancers. We evaluated parameters such as overall survival (OS), relapse-free survival (RFS), distant metastasis-free survival (DMFS), and the palliative performance scale (PPS) for the 37 targets. Based on significant *p*-values for these parameters, we selected 15 miR-660-5p target genes (Table 1, Supplementary Table 2).

**Table 1:** Potential targets of miR-660-5p

Gene Symbol	Full Name	Biological role
CALM1	Calmodulin 1	Participates in signaling pathways that modulates proliferation, motility and differentiation^39^
CDH13	Cadherin 13	Plays a critical role in tumor neovascularization, apoptosis, cell cycle, and cell proliferation 40
CDR2L	Cerebellar degeneration related protein 2 like	Member of the cerebellar degeneration related (CDR) protein family 41
CLEC3A	C-type lectin domain family 3 member A	Promotes cell differentiation, migration, and proliferation 42
FOLH1	Folate hydrolase (prostate-specific membrane antigen) 1	In the intestine, required for the absorption of folate 43. Involved in prostate tumor progression 44
KBTBD8	Kelch repeat and BTB domain containing 8	Promotes epithelial ovarian cancer progression 45
NR3C1	Nuclear receptor subfamily 3 group C member 1	Promotes proliferation and migration in clear cell renal cell carcinoma 46.
PGLYRP4	Peptidoglycan recognition protein 4	Bactericidal for Gram-positive and Gram-negative bacteria 47
PPP6R3	Protein phosphatase 6 regulatory subunit 3	Regulates cell cycle progression 48
RNF219	Ring finger protein 219	Overexpression promotes nasopharyngeal carcinoma cell proliferation, invasion, and migration 49
SLC46A3	Solute carrier family 46 member 3	Overexpression suppresses metastasis in hepatocellular carcinoma 50
TMEM41B	Transmembrane protein 41B	Host factor in virus inflections [26–28].
TPP2	Tripeptidyl peptidase II	Overexpression promotes cell growth, genetic instability, and resistance to apoptosis 51
USP53	Ubiquitin specific peptidase 53	Overexpression promotes metastasis in triple negative breast cancer 52
WDR36	WD repeat domain 36	Involved in cell cycle progression, apoptosis, signal transduction, and gene regulation 54, 53

**Fig. 3:**
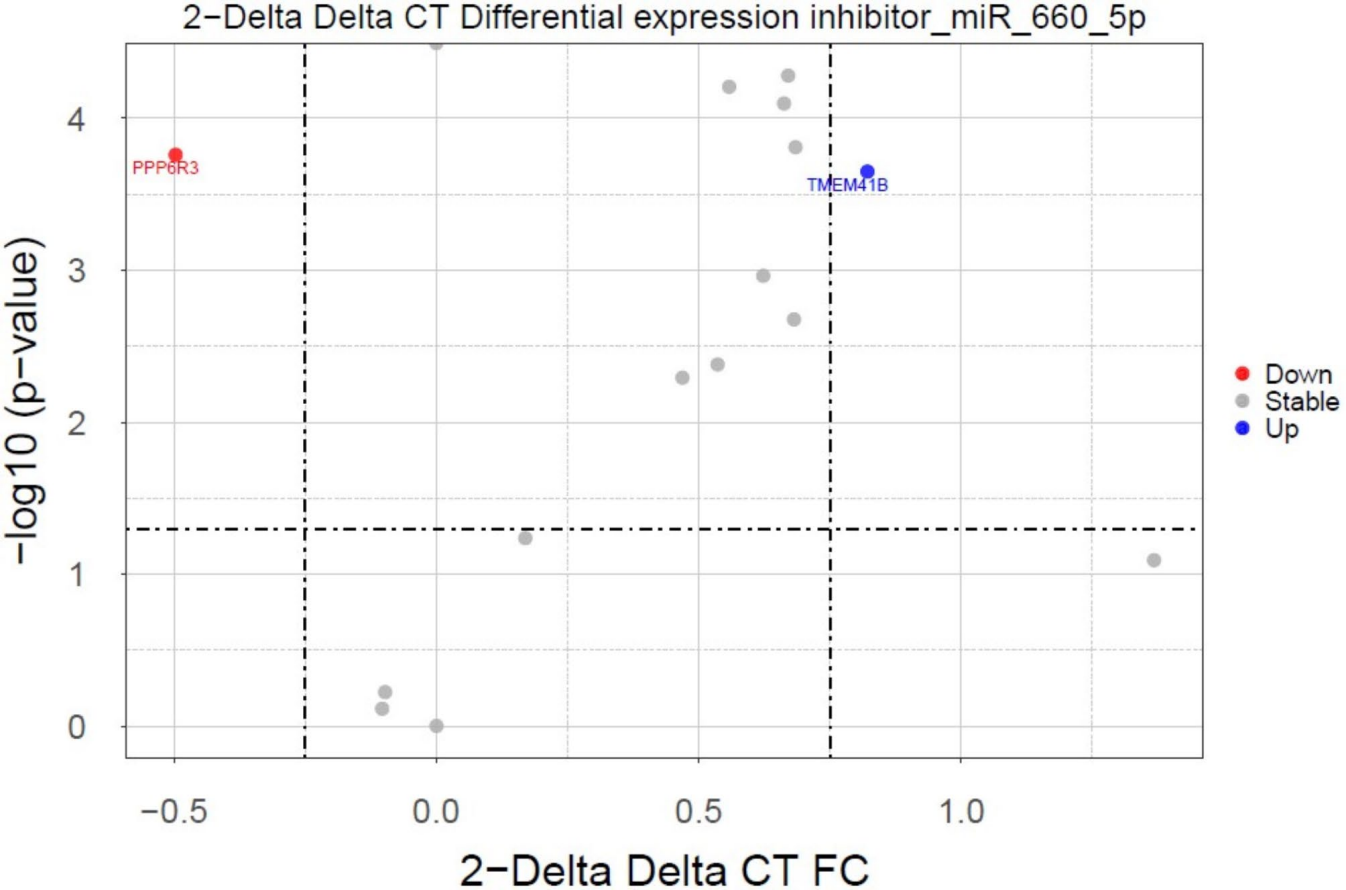
Validation of miR-660-5p target genes via RT‒qPCR. A volcano plot of the qPCR data showing changes in the mRNA expression of the potential targets identified through bioinformatic analysis in MDA-MB-231 cells. Gene expression was assessed on the basis of statistical significance and fold change, with a horizontal line at a *P*-value of 0.05 indicating significance and vertical lines delineating the (-0.25) and up (0.75) fold change thresholds. Gene status is represented by the following symbols: blue (TMEM41B) for upregulated genes, red (PPP6R3) for downregulated genes, and gray for stable genes

We subsequently conducted RT‒qPCR in MDA-MB-231 cells to measure changes in the expression of these 15 genes following miR-660-5p knockdown. Figure 3A shows a volcano plot illustrating the differential expression of potential miR-660-5p target genes. The horizontal line indicates genes significantly differentially expressed (*p* < 0.05) between the NC-miRNA inhibitor and miR-660-5p inhibitor conditions. The vertical lines denote the fold change thresholds for downregulated (-0.25) and upregulated (0.75) genes. In this plot, TMEM41B (in blue) was upregulated, whereas PPP6R3 (in red) was downregulated upon miR-660-5p knockdown. The remaining genes (in gray) either did not reach statistical significance or fell within the defined fold change thresholds, indicating stable expression levels. Nonetheless, although the other targets identified via RT‒qPCR did not significantly differ, they could still represent potential targets of miR-660-5p.

TMEM41B has been reported to be a tumor suppressor in prostate cancer [16]. TMEM41B, a transmembrane protein involved in the biogenesis of autophagosomes in the plasma reticulum, is required for phagophore maturation [17–19]. To understand the role of TMEM41B in breast cancer, we performed an Ingenuity Pathway Analysis (IPA) with all potential miR-660-5p targets analyzed via RT‒qPCR. The analysis of TMEM41B did not reveal any potential signaling pathways associated with breast cancer or any other process, and we found no prior reports in the literature regarding the role of this gene in breast cancer. On the other hand, some of the remaining potential miR-660-5p targets interact with the Akt and BRCA1 pathways, which are involved in key processes in breast cancer progression, such as proliferation, migration, and invasion (Supplementary Fig. 1).

### Validation of TMEM41B as a direct target of miR-660-5p in breast cancer cells

**Fig. 4:**
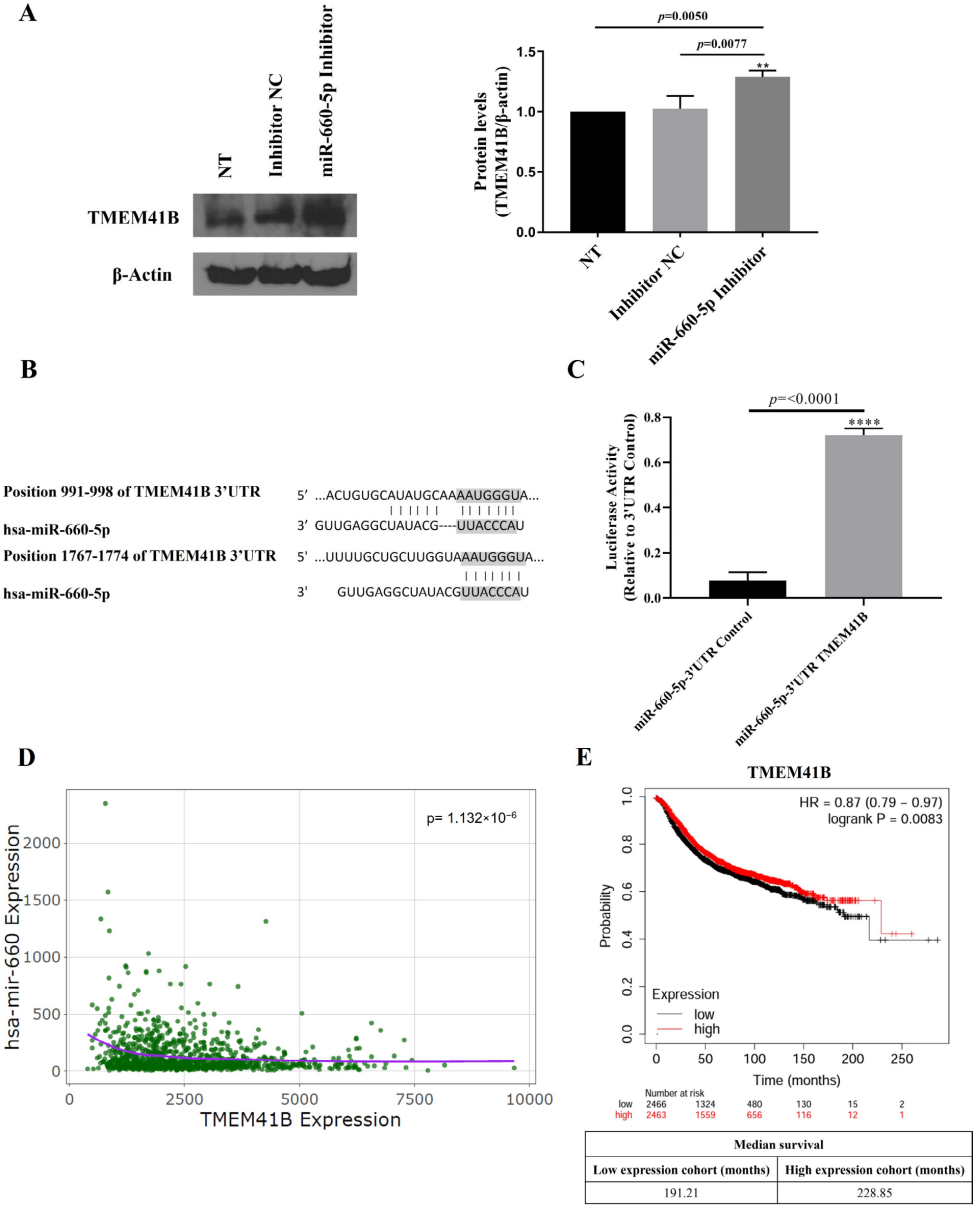
Validation of TMEM41B as a direct target of miR-660-5p in breast cancer cells. **(A)** Western blots showing the TMEM41B protein levels following the transfection of MDA-MB-231 cells with the miR-660-5p inhibitor. **(B)** Representation of the two regions where miR-660-5p is predicted to pair with TMEM41B, as identified by TargetScan. **(C)** In the dual-luciferase assay, MDA-MB-231 cells were transiently cotransfected with a miR-660-5p inhibitor and a TMEM41B 3´UTR-containing vector. The firefly luciferase activity was normalized to that of the Renilla control. The experiments were performed in triplicate. ** *p* < 0.01; **** *p* < 0.0001. **(D)** Correlation of miR-660 and TMEM41B expression. **(E)** TMEM41B expression-based relapse-free survival (RFS) analysis. K-M plots of breast cancer patients were generated via the K‒M plotter searchable database (www.kmplot.com). *P*-values < 0.05 were considered statistically significant

We then assessed the protein expression levels of TMEM41B following miR-660-5p knockdown. Our findings revealed a significant increase (*p* = 0.0077) in TMEM41B protein levels in MDA-MB-231 breast cancer cells compared with those in cells transfected with the NC-miRNA inhibitor (Fig. 4A). This result is consistent with the RT‒qPCR findings, which revealed that inhibiting miR-660-5p leads to an increase in TMEM41B expression. To confirm the direct interaction between miR-660-5p and the 3´ UTR of TMEM41B mRNA, we conducted dual-luciferase reporter assays. Two binding sites between miR-660-5p and TMEM41B were predicted via the online tool TargetScan (www.targetscan.org) (Fig. 4B). Cotransfection of the miR-660-5p inhibitor with the TMEM41B 3´UTR-containing vector in MDA-MB-231 cells resulted in a significant increase in luciferase activity compared with that of the control vector (*p* < 0.0001), indicating that miR-660-5p regulates TMEM41B expression by directly binding to its 3´UTR mRNA region (Fig. 4C). These findings provide robust evidence that TMEM41B mRNA is indeed a direct target of miR-660-5p in breast cancer cells.

The Spearman rank correlation coefficient (ρ\rho) was calculated as -0.1466, and together with the test statistic (S = 249,526,638), the corresponding *p*-value (1.132 × 10^− 6^) provided evidence to reject the null hypothesis (ρ\rho = 0) (Fig. 4D). These findings indicate that higher levels of hsa-miR-660 expression are associated with lower levels of TMEM41B expression, suggesting that their interaction may play a role in breast cancer tumor biology.

Based on the results presented in Supplementary Table 2, we plotted the relapse-free survival (RFS) data of patients stratified by TMEM41B expression. Survival curves revealed a significant correlation (*p* = 0.0083) between TMEM41B levels and RFS in breast cancer patients (*n* = 4929). Patients with lower TMEM41B levels had a median RFS of 191.21 months, whereas those with higher TMEM41B levels had a median RFS of 228.85 months (Fig. 4E). These findings underscore the prognostic value of both miR-660 and TMEM41B in breast cancer, highlighting their potential as biomarkers for patient stratification and therapeutic targeting.

## Discussion

In this study, we observed that miR-660-5p is significantly overexpressed in breast cancer cells compared with the human breast epithelial cell line MCF-10 A, which is consistent with findings of Shen et al. [13] and Peng et al. [14]. Elevated miR-660-5p levels have also been reported in breast cancer patients [9, 10], where it has been proposed as both a prognostic and diagnostic biomarker [10]. Unlike many miRNAs that exhibit tissue-specific functions as tumor suppressors or oncogenes [20], miR-660-5p is consistently reported as an oncomiR across various cancer types. It is overexpressed in non-small cell lung cancer [21]; lung cancer cell lines, including L9981-Luc; and metastatic human lung cancer cells [22], hepatocellular carcinoma [23], and chronic myeloid leukemia [24] cells.

The knockdown of miR-660-5p in breast cancer cells resulted in reduced cell proliferation, migration, invasion, and, unexpectedly, angiogenesis—an observation not previously reported. These effects were more pronounced for migration, invasion, and angiogenesis compared to cell proliferation in colony formation assays, suggesting that miR-660-5p regulated genes may be more closely associated with metastasis and angiogenesis. Similarly, it has been reported that miR-660-5p inhibition reduces proliferation, migration, and invasion in lung cancer cells [21], hepatocellular carcinoma cells [23], and osteosarcoma cells [25]. While in chronic myeloid leukemia cells miR-660-5p overexpression inhibits apoptosis [24].

Compared with previous reports, our study employed a comprehensive bioinformatic approach. Shen [13] and Peng [14] limited their search for miR-660-5p targets to a few databases, followed by experimental validation. We identified and validated TMEM41B as a target of miR-660-5p in breast cancer cells, which aligns with previous findings that miR-660-5p promotes breast cancer progression by targeting TET2 and activating the PI3K/AKT/mTOR pathway [14] and the miR-660-5p/TFCP2/CDKN1A pathway [13].

TMEM41B has been implicated in various pathologies, acting as a host factor in coronavirus and flavivirus infections [26–28] and as a tumor suppressor in prostate cancer [16]. In addition to TMEM41B, we identified LIFR among the potential targets of miR-660-5p (Supplementary Table 1), which has previously been reported as a tumor suppressor in breast cancer metastasis. Notably, detectable levels of LIFR expression are found only in non-metastatic breast cancer cell lines, such as SUM149, SUM159, MCF7, T47D, and SUM229, whereas in metastatic breast cancer cells, MDA-MB-231 and SUM1315 are not detectable [29]. Ectopic expression of LIFR in 4T1 and MDA-MB-231 metastatic cells have been shown to inhibit breast cancer cell invasion and migration. LIFR functions downstream of miR-9 and upstream of the Hippo–YAP signaling pathway [29]. These findings highlight the importance of validating the interaction between miR-660-5p and LIFR and exploring its role through functional assays. In contrast, most of the other potential targets of miR-660-5p, including VDAC1 [30], TPD52L2 [31], and YTHDF1 [32], play oncogenic roles in breast cancer. However, the roles of some other targets in breast cancer remain unknown.

The role of TMEM41B in breast cancer remains poorly understood. Future studies should investigate the molecular mechanisms and biological effects of TMEM41B overexpression or knockdown in breast cancer cells. Our findings did not conclusively determine whether one or both miR-660-5p target sites in the 3’ UTR of TMEM41B were responsible for the observed effect. Therefore, further investigations are needed to elucidate the molecular mechanism by which miR-660-5p regulates TMEM41B in breast cancer cells. In this context, additional experiments, such as site-directed mutagenesis of the target sites in luciferase reporter vectors, are recommended.

Notably, TMEM41B mutations have been reported in pulmonary carcinoid tumors. In this study, via IPA, TMEM41B was correlated with the NF-κB signaling pathway [33]. Dysregulation of NF-κB signaling promotes processes such as cell proliferation, metastasis, epithelial‒mesenchymal transition (EMT), and inflammation [34–36], which are critical in breast cancer progression. NF-κB is extensively involved in regulating breast cancer initiation, angiogenesis, and metastasis by increasing the expression of NF-κB-responsive genes [37, 38].

These findings underscore the complex interplay among miR-660-5p, TMEM41B, and NF-κB signaling in breast cancer, highlighting potential avenues for therapeutic intervention and further mechanistic exploration.

## Conclusions

In summary, the knockdown of miR-660-5p significantly reduces the proliferation, migration, and invasion of breast cancer cells and angiogenesis in HUVEC cells. These biological effects are possibly due to the post-transcriptional regulation of TMEM41B by this miRNA. Further exploration of the miR-660-5p/TMEM41B axis could provide valuable insights into its therapeutic potential and clinical relevance in breast cancer management.

## Electronic Supplementary Material

Below is the link to the electronic supplementary material.

Supplementary Material 1

Supplementary Material 2

Supplementary Material 3: Fig. 1. IPA analysis of potential miR-660-5p targets. (A) CLEC3A, (B) PGLYRP4, and (C) WDR36, KBTBD8, TPP2, FOLH1, PPP6R3, USP53, CALM1, and CDH13.

## Data Availability

No datasets were generated or analysed during the current study.

## Abbreviations

MiRNAs: microRNAs
HER2: Human epidermal growth factor receptor 2
TFCP2: Transcription factor CP2
TET2: Tet-eleven translocation 2
OS: Overall survival
RFS: Relapse-free survival
DMFS: Distant metastasis-free survival
PPS: Palliative performance scale
EMT: Epithelial‒mesenchymal transition
CALM1: Calmodulin 1
CDH13: Cadherin 13
CDR2L: Cerebellar degeneration related protein 2 like
CLEC3A: C-type lectin domain family 3 member A
FOLH1: Folate hydrolase (prostate-specific membrane antigen) 1
KBTBD8: Kelch repeat and BTB domain containing 8
NR3C1: Nuclear receptor subfamily 3 group C member 1
PGLYRP4: Peptidoglycan recognition protein 4
PPP6R3: Protein phosphatase 6 regulatory subunit 3
RNF219: Ring finger protein 219
SLC46A3: Solute carrier family 46 member 3
TMEM41B: Transmembrane protein 41B
TPP2: Tripeptidyl peptidase II
USP53: Ubiquitin-specific peptidase 53
WDR36: WD repeat domain 36
