# Supplementary material for: Inhibition of microRNA-660-5p decreases breast cancer progression through direct targeting of TMEM41B
Source: Hereditas. 2024 Dec 21;161:53. doi: 10.1186/s41065-024-00357-5 (PMC11662842; doi:10.1186/s41065-024-00357-5)
Supplement: Supplementary file 1 — Supplementary Material 1 [file 41065_2024_357_MOESM1_ESM.docx]

Supplementary information

**Supplementary Material 1: Table S1.** Selected miR-660-5p potential target genes as identified by bioinformatic analysis.

| **Gene Symbol** | **Full Name** | **Target Prediction** |
| --- | --- | --- |
| PPP6R3 | Protein phosphatase 6 regulatory subunit 3 | miRDB, RNACentral, DIANA, miRABEL |
| RNF219 | Ring finger protein 219 | TargetScan, miRDB, DIANA, miRABEL |
| NR3C1 | Nuclear receptor subfamily 3 group C member 1 | MiRDB, RNACentral, DIANA |
| CALM1 | Calmodulin 1 | TargetScan, mirRDB, DIANA, miRmap |
| LIFR | Leukemia Inhibitory Factor Receptor | MiRDB, RNACentral, DIANA, MicroT |
| ETV1 | ETS variant 1 | TargetScan, DIANA, miRmap |
| VDAC1 | Voltage dependent anion channel 1 | TargetScan, miRDB, miRABEL, MicroT |
| PRRG1 | Proline rich Gla (G-carboxyglutamic acid) 1 | TargetScan, miRmap, miRABEL |
| TPP2 | Tripeptidyl peptidase II | TargetScan, miRmap, miRABEL |
| HIF1A | Hypoxia inducible factor subunit Alpha | TargetScan, miRDB, miRABEL, MicroT |
| CD8A | CD8a molecule | TargetScan, miRDB, miRABEL, MicroT |
| ARL4C | ADP-ribosylation factor-like 4C | TargetScan, miRmap, miRABEL |
| CDH13 | Cadherin 13 | TargetScan, miRDB, miRABEL, MicroT |
| HAO1 | Hydroxyacid oxidase (glycolate oxidase) 1 | TargetScan, miRmap, miRABEL |
| JPH1 | Junctophilin 1 | miRDB, miRmap, miRABEL |
| CLEC3A | C-type lectin domain family 3 member A | TargetScan, miRDB, miRABEL |
| PGLYRP4 | Peptidoglycan recognition protein 4 | miRDB, miRmap, miRABEL |
| TPD52L2 | TPD52 like 2 | TargetScan, miRDB, miRmap, miRABEL, MicroT |
| SLC46A3 | Solute carrier family 46 member 3 | TargetScan, miRDB, miRmap, miRABEL |
| FOLH1 | Folate hydrolase (prostate-specific membrane antigen) 1 | TargetScan, miRmap, miRABEL, MicroT |
| DGKE | Diacylglycerol kinase épsilon | TargetScan, miRDB, miRmap, MicroT |
| KBTBD8 | Kelch repeat and BTB domain containing 8 | TargetScan, miRDB, miRmap, MicroT |
| USP53 | Ubiquitin specific peptidase 53 | miRDB, miSTAR, MicroT |
| APLP2 | Amyloid Beta Precursor Like Protein 2 | RNACentral, DIANA, miRABEL |
| YTHDF1 | YTH N6-Methyladenosine RNA Binding Protein 1 | DIANA, miRABEL, MicroT |
| CDR2L | Cerebellar degeneration retaled protein 2 like | MiRDB, miRABEL, MicroT |
| AOC3 | Amine oxidase, copper containing 3 | TargetScan, miRABEL, MicroT |
| EPAS1 | Endothelial PAS domain protein 1 | miRDB, miRABEL, MicroT |
| CNGA3 | Cyclic nucleotide gated channel Alpha 3 | miRDB, miRABEL, MicroT |
| IFT57 | Intraflagellar transport 57 homolog | TargetScan, miRABEL, MicroT |
| UNC79 | Protein Unc-79 Homolog | miRDB, miRmap, MicroT |
| KPNA4 | Karyopherin Subunit Alpha 4 | miRDB, miRmap, MicroT |
| KIF3A | Kinesin Family Member 3A | miRDB, miRmap, MicroT |
| TMEM41B | Transmembrane Protein 41B | TargetScan, miRmap, MicroT |
| WDR36 | WD Repeat Domain 36 | miRDB, miRmap, MicroT |
| TMED7-TICAM2 | TMED7-TICAM2 Readthrough | TargetScan, miRDB, MicroT |
| ARHGAP36 | Rho GTPase Activating Protein 36 | TargetScan, miRDB, MicroT |
