# Supplementary material for: Inhibition of microRNA-660-5p decreases breast cancer progression through direct targeting of TMEM41B
Source: Hereditas. 2024 Dec 21;161:53. doi: 10.1186/s41065-024-00357-5 (PMC11662842; doi:10.1186/s41065-024-00357-5)
Supplement: Supplementary file 2 — Supplementary Material 2 [file 41065_2024_357_MOESM2_ESM.docx]

Supplementary information

**Supplementary Material 2: Table S2.** Kaplan-Meier plotter analysis of miR-660-5p potential target genes

| **Gene Symbol** | **Kaplan-Meier Plotter Analysis (*p*-values)** | | | |
| --- | --- | --- | --- | --- |
|  | **Overall survival (OS)** | **Relapse-free survival (RFS)** | **Distant metastasis-free survival (DMFS)** | **Palliative performance scale (PPS)** |
| PPP6R3 | 0.0051 | 0.81 | 0.3051 | 0.0314 |
| RNF219 | 0.38 | 0.18 | 0.8061 | 0.0026 |
| NR3C1 | 0.042 | 0.81 | 0.0021 | 0.7143 |
| CALM1 | 0.013 | 0.00059 | 0.224 | 0.4456 |
| LIFR | 0.00013 | 1.60E-08 | 0.0078 | 0.151 |
| ETV1 | 0.0909 | 0.0001 | 0.1536 | 0.9739 |
| VDAC1 | 0.0227 | 7.6E-07 | 0.6867 | 0.1437 |
| PRRG1 | 0.0027 | 1.70E-06 | 4.9E-07 | 0.0656 |
| TPP2 | 0.0271 | 0.0415 | 0.0022 | 0.6922 |
| HIF1A | 1.90E-06 | 1.60E-10 | 0.016 | 2.80E-07 |
| CD8A | 0.0028 | 0.0002 | 0.7163 | 0.327 |
| ARL4C | 0.35 | 4.7E-05 | 0.075 | 0.14 |
| CDH13 | 0.3773 | 0.3964 | 0.6696 | 0.0319 |
| HAO1 | 0.904 | 0.0032 | 0.5184 | 0.2595 |
| JPH1 | 0.0093 | 0.1239 | 0.0757 | 0.8703 |
| CLEC3A | 0.1199 | 0.2589 | 0.9038 | 0.0594 |
| PGLYRP4 | 0.7101 | 0.0005 | 0.0277 | 0.3224 |
| TPD52L2 | 0.0025 | 1.00E-07 | 0.1497 | 0.1516 |
| SLC46A3 | 0.6354 | 0.0041 | 0.557 | 0.2489 |
| FOLH1 | 0.1605 | 0.0011 | 0.0406 | 0.3045 |
| DGKE | 0.2803 | 7.70E-10 | 0.0105 | 0.9116 |
| KBTBD8 | 0.0048 | 0.0053 | 0.5069 | 0.0678 |
| USP53 | 0.0092 | 0.4283 | 0.0471 | 0.9109 |
| APLP2 | 0.0002 | 0.738 | 0.1171 | 0.936 |
| YTHDF1 | 0.0533 | 0.2133 | 0.0424 | 0.7076 |
| CDR2L | 0.029 | 0.0019 | 0.0146 | 0.0461 |
| AOC3 | 0.0076 | 0.8773 | 0.9634 | 0.1099 |
| EPAS1 | 0.0417 | 0.829 | 0.2997 | 0.0754 |
| CNGA3 | 0.0097 | 0.8562 | 0.0844 | 0.7623 |
| IFT57 | 5.7E-05 | 0.0446 | 0.0979 | 0.9381 |
| UNC79 | 0.044 | 0.6376 | 0.0731 | 0.7425 |
| KPNA4 | 0.0011 | 0.8685 | 0.0191 | 0.863 |
| KIF3A | 3.80E-06 | 0.1906 | 0.0069 | 1.10E-06 |
| TMEM41B | 0.0634 | 0.0083 | 0.0377 | 0.5128 |
| WDR36 | 0.0366 | 0.0425 | 0.3323 | 0.0053 |
| TMED7-TICAM2 | 0.0553 | 0.2019 | 9.30E-05 | 0.9404 |
| ARHGAP36 | 0.0002 | 0.0027 | 0.0008 | 0.7725 |
