# Supplementary figures and images for: Inhibition of microRNA-660-5p decreases breast cancer progression through direct targeting of TMEM41B
Source: Hereditas. 2024 Dec 21;161:53. doi: 10.1186/s41065-024-00357-5 (PMC11662842; doi:10.1186/s41065-024-00357-5)

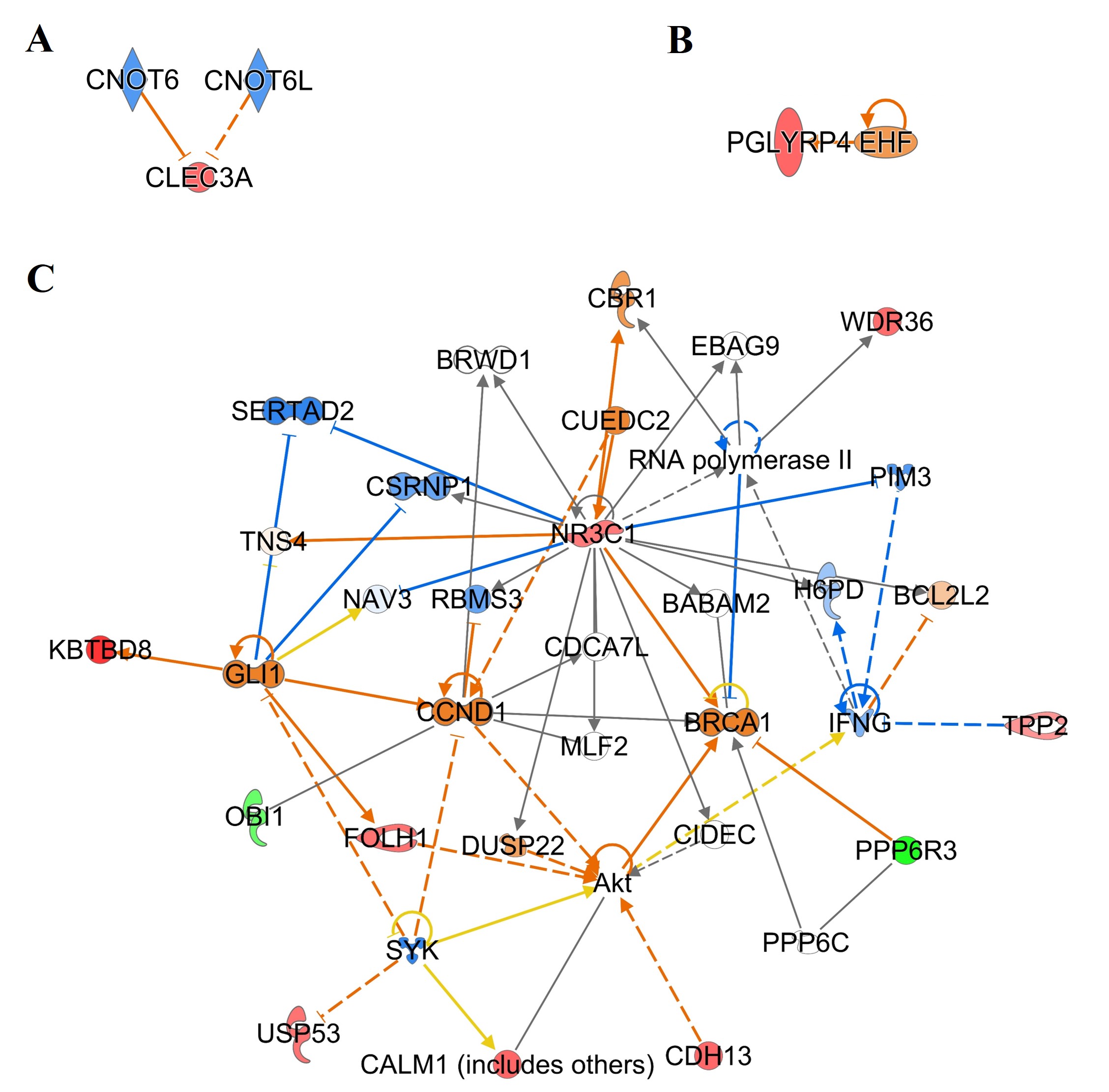

Supplement: Supplementary file 3 — Supplementary Material 3: Fig. 1. IPA analysis of potential miR-660-5p targets. (A) CLEC3A, (B) PGLYRP4, and (C) WDR36, KBTBD8, TPP2, FOLH1, PPP6R3, USP53, CALM1, and CDH13. [file 41065_2024_357_MOESM3_ESM.jpg]
